# Ostéome ostéoïde intra-articulaire de la hanche: deux observations et revue de la littérature

**DOI:** 10.4314/pamj.v8i1.71051

**Published:** 2011-01-31

**Authors:** Ellouz Zoubir, Faten Frikha, Moez Trigui, Wajdi Bouaziz, Lobna Ayedi, Mourad Aoui, Fakher Gdoura, Chaouki Dabbeh, Zouhir Bahloul, Tahia Boudawara, Kamel Ayedi, Hassib Keskes

**Affiliations:** 1 Service d’Orthopédie et Traumatologie CHU Habib Bourguiba 3029 Sfax, Tunisie; 2 Service de Médecine interne CHU Hedi Chaker 3029 Sfax, Tunisie; 3 Laboratoire d’anatomopathologie CHU Habib Bourguiba 3029 Sfax Tunisie; 4 Service de radiologie CHU Habib Bourguiba 3029 Sfax Tunisie

## Abstract

Abstract L’ostéome ostéoïde est une tumeur osseuse bénigne qui affecte les adultes jeunes et se localise préférentiellement au niveau des os longs. La localisation intra-articulaire est rare et atteint le plus souvent la hanche. La symptomatologie clinique est alors atypique et peut faire errer le diagnostic constituant un défi diagnostique pour les cliniciens. Nous rapportons deux observations d’ostéome ostéoïde intra-articulaires de la hanche chez deux hommes âgés 24 et 45 ans, révélés par des douleurs de la hanche gauche de type inflammatoire évoluant depuis un an et un an et demi respectivement. Chez les deux patients, le tableau atypique de l’ostéome ostéoïde a été à l’origine d’un retard diagnostic. La tomodensitométrie est dans cette indication l’examen le plus spécifique qui a permis d’évoquer le diagnostic d’ostéome ostéoïde. Une fois le diagnostic est posé, l’exérèse chirurgicale à ciel ouvert a permis la guérison avec disparition totale des douleurs. L’examen histologique a confirmé le diagnostic final d’ostéome ostéoïde intra-articulaire dans les deux cas.

## Introduction

L’ostéome Ostéoïde (OO) est une tumeur ostéoblastique bénigne relativement fréquente. Il survient généralement chez l’adulte jeune et se localise préférentiellement sur la diaphyse et la métaphyse des os longs [1]. La localisation intra-articulaire est rare représentant environ 10 à 13 % des cas [1,2]. L’articulation le plus souvent atteinte est la hanche [3]. L’OO intra-articulaire revêt généralement une clinique atypique pouvant faire retarder le diagnostic. Cela est vrai surtout pour une articulation profonde comme la hanche. Nous rapportons ici deux observations d’OO intraarticulaire et nous essayons de décrire les particularités clinico-radiologiques et les modalités thérapeutiques de cette localisation.

## Patients et observations

**Observation 1**

Un jeune homme de 24 ans, sans antécédents notables, présente depuis 12 mois des douleurs intermittentes de la hanche gauche à recrudescence nocturne, apparues sans facteurs déclenchant. Ces douleurs sont légèrement calmées par les antalgiques et les anti-inflammatoires non stéroïdiens.

**Figure 1:**
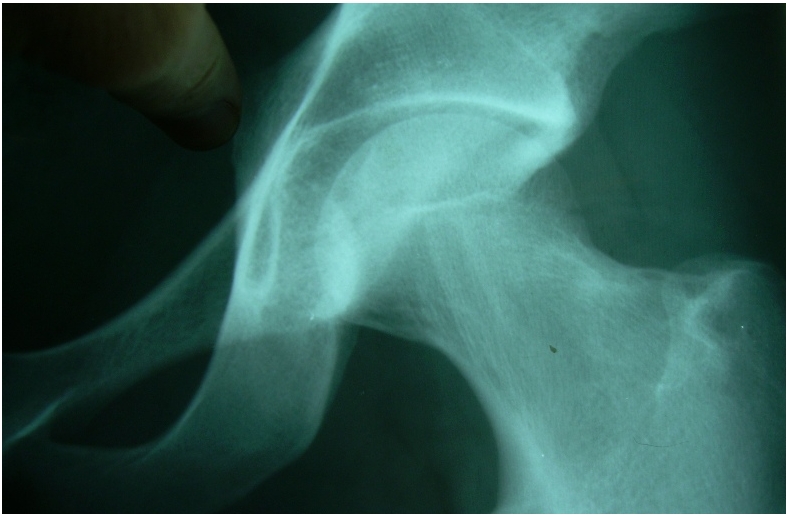
Radiographie de la hanche gauche. Ostéocondensation du bord supérieur du col du fémur gauche

L’examen clinique trouve une limitation importante et douloureuse des mouvements de la hanche gauche. L’état général est bon. L’hémogramme et la vitesse de sédimentation sont normaux. Les radiographies de la hanche et du bassin montrent une discrète ostéocondensation du bord supérieur du col du fémur gauche (Figure 1).

**Figure 2:**
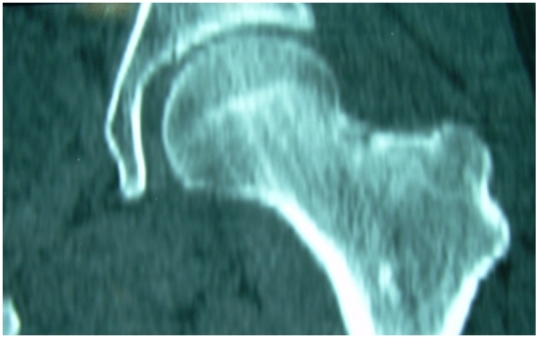
Tomodensitométrie de la hanche gauche qui visualise le nidus de l’ostéome ostéoïde avec une effraction du bord supérieur du col fémoral

La tomodensitométrie objective l’image typique d’un nidus avec une effraction du bord supérieur du col fémoral faisant évoquer le diagnostic d’ostéome ostéoïde (OO) intra-articulaire de la hanche (Figure 2).

**Figure 3:**
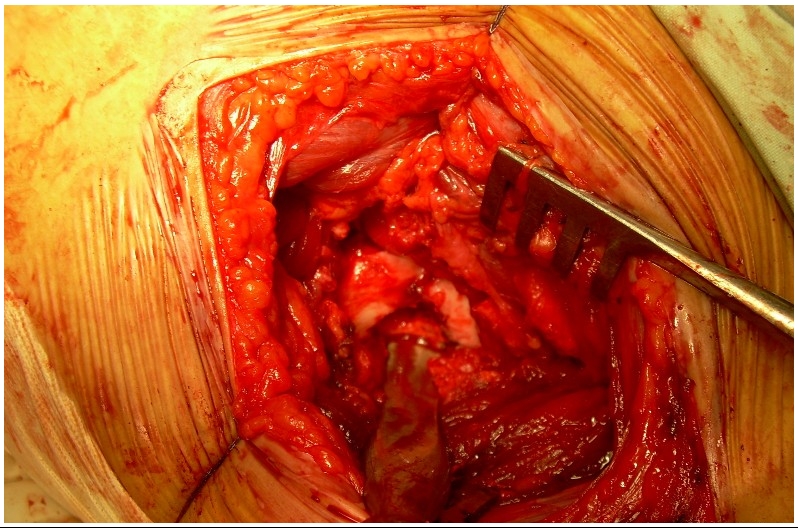
Vue opératoire. Arthrotomie de la hanche par voie antérieure - exérèse du nidus

**Figure 4:**
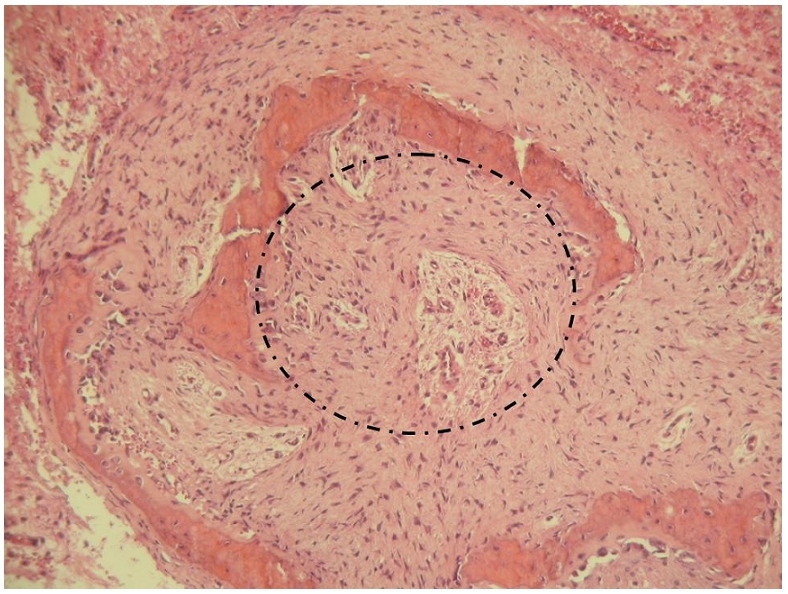
Examen anatomopathologique de la pièce d’exérèse. Prolifération richement vascularisée du nidus entourée de travées ostéoïdes riches en cellules ostéoblastiques

L’arthrotomie de la hanche par voie antérieure permet de voir le nidus affleurer à travers la corticale supérieure du col fémoral. L’exérèse du nidus et de l’os condensé au tour est alors aisée (Figure 3). L’examen anatomopathologique de la pièce d’exérèse confirme alors le diagnostic d’OO, retrouvant en périphérie du nidus des travées ostéoïdes riches en cellules ostéoblastiques (Figure 4). L’appui est interdit pour une durée de 45 jours. Les suites opératoires sont simples avec disparition immédiate des douleurs et guérison complète.

**Observation 2**

Un homme, âgé de 45 ans, consulte pour une douleur de la hanche gauche ayant débuté environ 1 an et demi auparavant, qu’il se dit secondaire à un traumatisme.

Cette douleur est de type inflammatoire avec recrudescence nocturne, partiellement calmée par les antalgiques et les anti-inflammatoires non stéroïdiens. On note alors à l’examen une boiterie à la marche, la mobilité de la hanche est limitée avec des douleurs provoquées très importantes. Le reste de l’examen est normal. L’état général est conservé. Le bilan inflammatoire est sans anomalies par ailleurs. Les radiographies standard de la hanche ne montrent aucune lésion osseuse.

**Figure 5:**
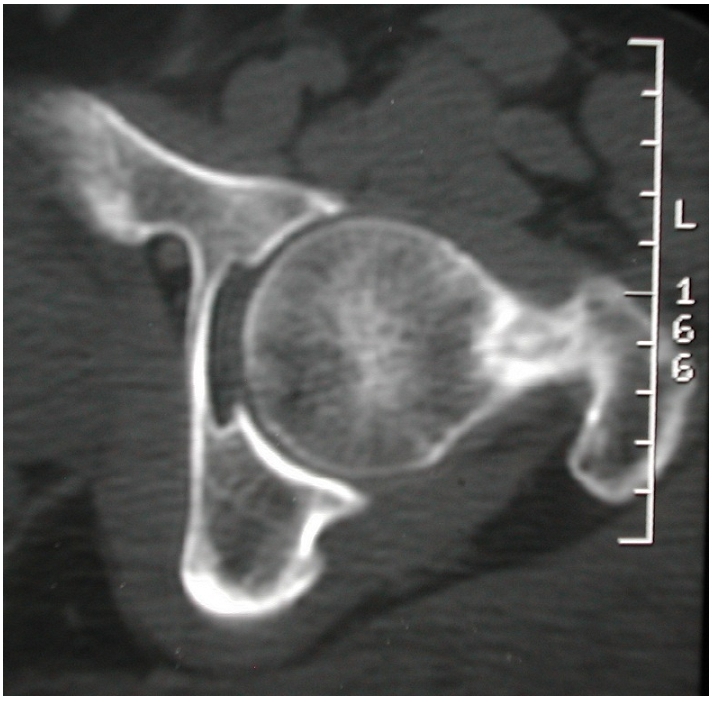
Tomodensitométrie de la hanche gauche. Ostéome ostéoïde intra articulaire du col du fémur - petite formation lytique au sein de laquelle se trouve un nidus dense entouré d’un halo clair

Plusieurs diagnostics ont été évoqués: algodystrophie de la hanche, ostéonécrose aseptique de la tête fémorale ou alors monoarthrite. L’exploration est complétée par une scintigraphie osseuse qui montre une hyperfixation intense non spécifique de la hanche. L’IRM montre des signes évoquant une algodystrophie avec des anomalies du signal de l’extrémité supérieure du fémur gauche (Hypo T1, hyper T2). C’est la tomodensitométrie en coupes fines qui va permettre d’objectiver l’ostéome ostéoïde devant l’image en cocarde du nidus (Figure 5).

**Figure 6:**
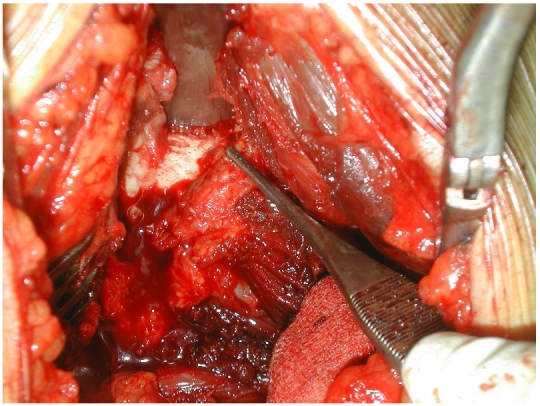
Vue opératoire de l’ostéome ostéoïde de la hanche gauche: le nidus apparaît au niveau de la face supérieure du col fémoral

Il a été décidé de réaliser une exérèse chirurgicale avec ablation complète de la tumeur. La hanche a été abordée par voie antérieure. La capsule articulaire était bombée témoignant d’un épanchement articulaire. L’arthrotomie antérieure a permis de confirmer l’épanchement et de l’évacuer, de constater une hypertrophie de la synoviale articulaire et de voir le nidus au niveau supérieur de la base du col fémoral. Le nidus et l’os condensé l’entourant ont été réséqués (Figure 6). Il n’a pas été fait d’ostéosynthèse préventive du col mais l’appui a été interdit pour 45 jours.

L’examen anatomopathologique du produit d’exérèse a permis de conclure à un OO. La symptomatologie douloureuse et la limitation de la mobilité articulaire ont complètement disparues à la suite de l’intervention.

## Discussion

L’ostéome ostéoïde (OO) est une tumeur osseuse primitive bénigne fréquente. Il représente 2 à 3% de l’ensemble des tumeurs osseuses et 10 à 20 % de l’ensemble des tumeurs osseuses bénignes [4,4-6].

Il se situe préférentiellement au niveau des os longs [1,7] avec une prédilection pour les membres inférieurs [8], notamment le tibia et le fémur. La localisation intra-articulaire est rare et sa fréquence est difficile à apprécier, environ 10 à 13 % des cas [1-3,9]. Il touche alors principalement la hanche comme nos deux observations [2,3,6,10-12] mais aussi le genou [9,13], le coude [14,15], le poignet et le carpe [5,8,16,17].

Comme retrouvé dans nos deux observations, les patients atteints d’OO sont dans la majorité des cas des hommes dont la douleur se révèle entre l’âge de 15 et 35 ans [5].

Les manifestations cliniques de l’OO sont le plus souvent faites de douleurs nocturnes, insomniantes, calmées par la prise de salicylés [8,17]. Cependant, un OO intra-articulaire (OOIA) évolue fréquemment dans un contexte trompeur [6] retardant le diagnostic et la prise en charge adéquate [6]. Les symptômes cliniques les plus communs décrits au cours des OOIA sont des douleurs articulaires, des synovites, une raideur ou une tuméfaction des parties molles et une diminution des mobilités articulaires [18]. L’examen clinique est peu spécifique notamment pour les articulations profondes et plusieurs autres diagnostics dans ces localisations peuvent être évoqués : l’ostéonécrose aseptique, l’algodystrophie, les arthrites rhumatismales ou infectieuses notamment tuberculeuses et l’ostéochondrite [2,9,13]. Dans notre deuxième observation, la présence d’une symptomatologie clinique atypique associée à l’absence de lésion à la radiologie conventionnelle et les atypies retrouvées à la scintigraphie osseuse ont fait suspecter une algodystrophie ou une ostéonécrose aseptique de la tête fémorale.

Dans la littérature, les délais diagnostiques des OO intra-articulaires s’étendent de 4 mois à 5 ans [2,3,6,9], plus importants que dans les autres localisations. Dans nos deux observations, le délai diagnostique a été plus que 2 mois.

Les OO sont le plus souvent diagnostiqués par les simples rayons X ou le scanner [19] qui montrent une image lytique centrale de petite taille correspondant au nidus entourée d’une sclérose réactionnelle [17].

Dans sa localisation intra-articulaire, l’image typique du nidus bordée d’une ostéosclérose périphérique est absente dans la majorité des cas [9]. La radiographie est soit normale, comme chez notre 2ème patient, soit montre une ostéopénie péri-articulaire [2,3]. La sclérose réactionnelle peut masquer le nidus radiotransparent et peut s’associer à une déminéralisation pouvant en imposer pour une algodystrophie [5]. Au niveau de la hanche, cette ostéoporose régionale est fréquente [20] et pour les patients dont les douleurs évoluent depuis au moins 3 mois, un élargissement du col du fémur peut être observé [20].

Toutes ces particularités cliniques et radiologiques des OO intra-articulaires imposent le recours fréquent à plusieurs moyens d’imagerie [3]. La scintigraphie osseuse, qui garde une place parmi les différents moyens diagnostics avec une sensibilité atteignant les 100% [19], révèle une fixation localisée « en spot » précoce et intense [4]. Elle ne permet pas de confirmer le diagnostic positif [14], mais son intérêt réside de rechercher d’autres localisations généralement exceptionnelles [16, 19] et sert à cibler précisément le reste du bilan d’imagerie (TDM et IRM) [7]. L’IRM représente pour certains auteurs l’examen le plus sensible pour porter le diagnostic d’OO [21]. Elle retrouve le nidus, et montre fréquemment un oedème intra-osseux et des parties molles périlésionnelles [21]. Cependant l’IRM manque de spécificité, et le nidus peut ne pas être visualisé dans près de 50 % des cas [2]. Le scanner représente l’examen de référence le plus spécifique pour le diagnostic d’OO lorsque les radiographies sont peu contributives [4,6]. Il permet de localiser avec précision la lésion, de mesurer la dimension exacte du nidus et d’évaluer son extension locale ce qui permet d’orienter la stratégie thérapeutique [8,19]. Nos deux observations ont pour intérêt de préciser l’apport de la tomodensitométrie afin de diagnostiquer de façon précoce les OOIA. Seul cet examen a pu mettre en évidence l’image caractéristique du nidus.

Dans la littérature, le traitement de choix de cette tumeur bénigne, bien qu’elle puisse involuer spontanément après des années, est l’exérèse chirurgicale qui doit être complète pour éviter les récidives. La résection en bloc est la technique qui permet la résection en totalité du nidus [8] et donc la guérison. Elle peut être obtenue par chirurgie classique à ciel ouvert ou par techniques plus modernes mini-invasives en percutanée après repérage du nidu par broche sous contrôle tomodensitométrique [22]. Depuis les années 1990, ces techniques mini-invasives prennent une place importante dans l’arsenal thérapeutique des OO particulièrement la thermo ablation par radiofréquence [1,12,22] dont le taux de succès est de 70 à 100% [23,24]. L’inconvénient de ces techniques de destruction du nidus est l’absence de possibilité d’examen anatomopathologique. Dans les localisations intra-articulaires ou à proximité d’une articulation, dont l’accès est parfois difficile, certains auteurs proposent l’exérèse par voie arthroscopique [5,10]. Nos deux patients ont bénéficié d’une exérèse monobloc à ciel ouvert dont les suites ont été marquées par une bonne évolution clinique. Cette chirurgie a permis d’obtenir en plus une confirmation histologique souvent nécessaire pour le diagnostic et pour confirmer le caractère complet de l’exérèse.

## Conclusion

Les formes intra-articulaires des ostéomes ostéoïdes sont rares et leur diagnostics est le plus souvent difficile facilité par l’apport des techniques d’imagerie médicales. En cas de doute diagnostic, la tomodensitométrie représente l’examen le plus spécifique permettant le diagnostic positif. L’exérèse chirurgicale complète de la lésion permet le plus souvent la guérison totale et évite les récidives.

## Conflits d’intérêt

Les auteurs ne déclarent aucun conflit d’intérêts.

## Contribution des auteurs

Tous les auteurs ont contribué a la prise en charge du patient et la rédaction du manuscrit.
